# Design, Synthesis and Evaluation of New Marine Alkaloid-Derived Pentacyclic Structures with Anti-Tumoral Potency

**DOI:** 10.3390/md13010655

**Published:** 2015-01-19

**Authors:** Sebastien Boucle, Celine Melin, Marc Clastre, Jerome Guillard

**Affiliations:** 1 Center for AIDS Research, Laboratory of Biochemical Pharmacology, Department of Pediatrics, Emory University School of Medicine, and Veterans Affairs Medical Center, Decatur, GA 30033, USA; E-Mail: s.boucle@hotmail.fr; 2 University François Rabelais de Tours, EA2106 Biomolécules et Biotechnologies Végétales, 31 avenue Monge, 37200 Tours, France; E-Mails: celine.melin@univ-tours.fr (C.M.); marc.clastre@univ-tours.fr (M.C.); 3 University of Poitiers, UMR CNRS IC2MP 7285, 40 avenue du Recteur Pineau, 86022 Poitiers Cedex, France

**Keywords:** makaluvamine, pyrroloiminoquinone, marine drugs analogues, topoisomerase II inhibitor, antitumor activity

## Abstract

This work describes the synthesis and biological evaluation of a new heterocyclic hybrid derived from the ellipticine and the marine alkaloid makaluvamine A. Pyridoquinoxalinedione **12** was obtained in seven steps with 6.5% overall yield. **12** and its intermediates **1**–**11** were evaluated for their *in vitro* cytotoxic activity against different cancer cell lines and tested for their inhibitory activity against the human DNA topoisomerase II. The analysis by electrophoresis shows that the pentacycle **12** inhibits the topoisomerase II like doxorubicine at 100 µM. Compound **9** was found to have an interesting profile, having a cytotoxicity of 15, 15, 15 and 10 μM against Caco-2, HCT-116, Pc-3 and NCI cell lines respectively, without any noticeable toxicity against human fibroblast.

## 1. Introduction

**Figure 1:**
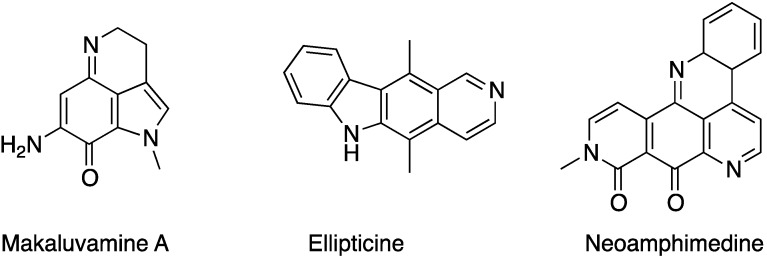
Alkaloids with anti-topoisomerase II activity.

Research leading to the characterization of naturally occurring secondary metabolites continues to offer one of the most significant pathways contributing to drug discovery [1]. New leads for the preclinical development of therapeutic agents are often inspired by natural products, which engage initial interest owing to reports of their biological activity [2]. Indeed, the unique molecular architecture of these natural substances may serve as primary stimulus for innovation and creativity to provide precisely engineered chemical solutions for complex biological phenomena. Among secondary metabolites is found a class of compounds called alkaloids [3,4,5]. This concept, introduced by Meissner [6] from the latin *alkali* (basic) regroups many chemical structures that are important sources of bioactive compounds, such as ellipticine [7], neoamphimedine, isolated from *xestospongia* sponge [8] or makaluvamine A [9] (Figure 1), extracted from another marine sponge. In fact, marine biodiversity holds an exceptional number of species. So far, more than 300,000 marine species have now been discovered; some specialists agree that over than a million are still to be discovered [10,11]. Such diversity leaves a space wide-open for new discoveries, presenting the marine world as a great reservoir for new active drugs.

Between 2001 and 2010, most of the compounds isolated from marine sources were alkaloids or macrocycles, and they have been isolated from sponges [12,13]. From the *Latrunculiidae* sponge family have been extracted some pyrroloiminoquinones [14,15,16], an important class of bioactive alkaloids. This class of metabolites includes discorhadbines and makaluvamines isolated primarily from sponges of the *Zyzzya* genus. They have been reported to express significant cytotoxic activity towards several tumor cells, as well as a potential activity against the topoisomerase II [17].

Our goal is to study the synthesis and the biological evaluation of new pyridoquinoxalinediones as potential intermediates for the synthesis of new analogues of makaluvamine A, along with pentacyclic structures as potential anti-topoisomerase II agents. 

## 2. Results and Discussion

### 2.1. Analogue Design and Chemistry 

**Scheme 1:**
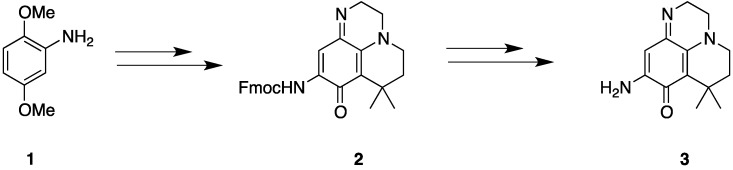
Synthesis of the aminopyridoiminoquinone **8**.

As previously reported by our research group, the 10 steps of the synthesis of compound **3** were described (Scheme 1), starting from 2,5-dimethoxyaniline **1** [18].

**Scheme 2:**
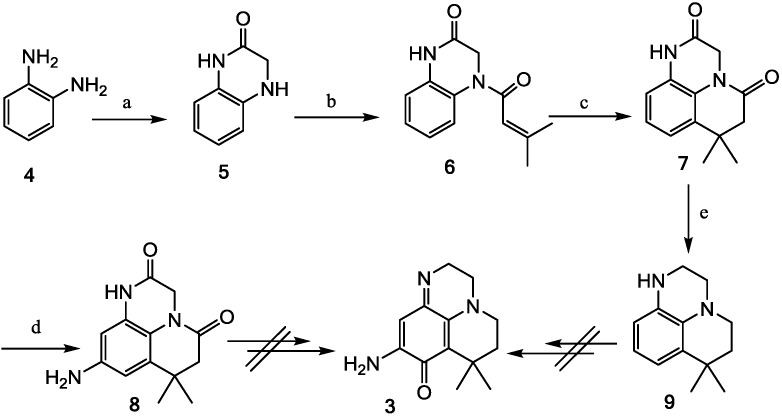
Synthesis of the aminopyridiniminoquinone **3**. (**a**): Bromoacetylbromide, THF, Et_3_N, reflux, 2 days, 65%; (**b**): dimethylacryloylchloride, pyridine, 2h, 78%; (**c**): (i) AlCl_3_, DCM, 24 h, 83%; (**d**): (i) HNO_3_, DCM/DMF, 24 h, 42%; (ii) H_2_, Pd/C, DMF/DMSO, 18 h, 70%; (**e**): BH_3_/THF, THF, 2 h, reflux, 81%.

In order to rapidly access new analogues, we have developed a new approach that could lead to the same compound in only seven steps, starting from 1,2-benzenediamine **4** (Scheme 2). Dihydroquinoxalinone **5** was obtained with 66% yield after condensation of dimethylacryloylchloride [19]. The tricyclic intermediate **7** was then synthesized in two steps with a 65% yield, after acylation conducted in pyridine and cyclisation using aluminum chloride in dichloromethane. The nitration of the tricyclic compound was achieved using fuming nitric acid in dichloromethane in a 42% yield, which could be easily reduced by catalytic hydrogenation leading to the amino intermediate **8** with a 70% yield. Unfortunately, oxidation to the desired iminoquinone using Fremy salts or cerium ammonium nitrate was never obtained. Reduction of pyridoquinoxalinedione **7** using boran/tetrahydrofuran reagent yielded compound **9**, which could not be oxidized in order to reach the desired pyridoiminoquinone **3**.

**Scheme 3:**
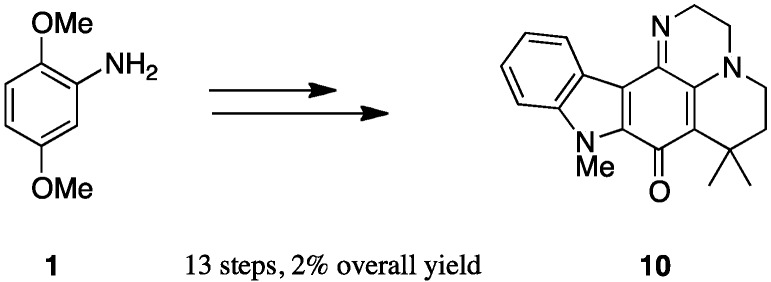
Synthesis of pentacycle **10**.

We have recently described the synthesis of a pentacyclic hybrid molecule **10** (Scheme 3) of makaluvamine A and ellipticine [20]. 

**Scheme 4:**
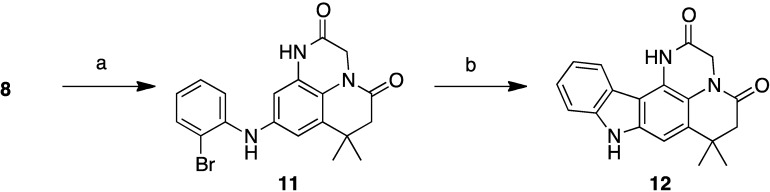
Synthesis of pentacycle **12**. (**a**): 2-Bromoiodobenzene, Pd_2_dba_3_, Xantphos, K_2_CO_3_, dioxane, schlenk, 18 h, 71%; (**b**): (i) Pd(OAc)_2_, Pcy_3_HBF_4_, K_2_CO_3_, DMA, reflux, 24 h, sealed tube, 40%.

As we had great interest in these polycylic structures with a topoisomerase II inhibition potential, we have developed a two-step pathway to the pentacyclic analogue compound in this series from intermediate **8** (Scheme 4). The Buchwald-Hartwig coupling between **8** and the 2-bromoiodobenzene led to compound **11** with 71% yield according to our previously reported procedure [20]. The cyclisation led to the desired pentacyclic structure **12** through the Heck reaction, using palladium acetate and potassium carbonate in dimethylacetamide in a sealed tube.

All synthesized compounds were then biologically evaluated in order to determine their potential against different cancer cell lines, as well as their topoisomerase II inhibition efficiency. 

### 2.2. Bioactivities of the New Synthesized Analogues 

Synthesized molecules were tested against different cancer cell lines in order to evaluate their antitumoral potency. In a second stage we will describe the anti-topoisomerase II efficiency in order to determine the mechanism of action of these new synthetic analogues of marine alkaloids.

#### 2.2.1. *In Vitro* Cytotoxicity

All synthesized compounds were evaluated for their *in vitro* cytotoxic activity against different human cancer cell lines, Caco-2, HCT-116, HUH-7, MDA-MB-231, Pc-3 and NCI. A human fibroblast cell line was used as control for the overall toxicity. Compounds were tested up to 25 μM (as shown in Table 1) for compounds with any activity at <25 μM. All other tested compounds were found to be inactive against these cell lines at 25 μM.

**Table 1:** Cytotoxicity of the synthesized molecules, IC_50_ expressed in μM.

Cmpd	IC_50_ in μM						
	HUH-7	CaCo-2	MDA-MB-231	HCT-116	Pc-3	NCI	Fib. Hum.
**9**	20	15	20	15	15	10	>25
**11**	20	20	>25	25	25	>25	25
**12**	20	20	20	10	6	10	6 (50%)

#### 2.2.2. Topoisomerase II inhibition

**Figure 2:**
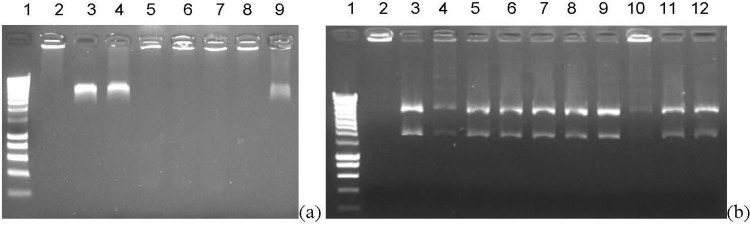
Topoisomerase II decatenation of kinetoplast-DNA (kDNA) assay. (**a**) doxorubicine (doxo) control: (1) size marker; (2) kDNA only; (3) kDNA + TopoII; (4) kDNA + TopoII + DMSO; (5) kDNA + TopoII + doxo 200 μM; (6) kDNA + TopoII + doxo 100 μM; (7) kDNA + TopoII + doxo 75 μM; (8) kDNA + TopoII + doxo 50 μM; (9) kDNA + TopoII + doxo 10 μM; (**b**) synthesized compounds test (1) size marker; (2) kDNA only; (3) kDNA + TopoII; (4) kDNA + TopoII + doxo 50 μM; (5) kDNA + TopoII + cmpd **1** 100 μM; (6) kDNA + TopoII + cmpd **2** 100 μM; (7) kDNA + TopoII + cmpd **3** 100 μM; (8) kDNA + TopoII + cmpd **9** 100 μM; (9) kDNA + TopoII + cmpd **7** 100 μM; (10) kDNA + TopoII + cmpd **12** 100 μM; (11) kDNA + TopoII + cmpd **11** 100 μM; (12) kDNA + TopoII + cmpd **8** 100 μM.

Inhibition activity of compounds **1**–**12** was evaluated against human DNA topoisomerases II. Synthesized molecules were screened at 100 μM for anti-topoisomerase II activity. Figure 2 indicates that compound **12** inhibits the human DNA topoisomerase II as well as doxorubicine at 100 μM. Other synthesized compounds surprisingly did not express any inhibition of the topoisomerase [20]. 

## 3. Experimental Section 

### 3.1. Biology

#### 3.1.1. Cytotoxicity Assay

Cytotoxicity assays of compounds **1**–**12** were performed using the MTT [3-(4,5-dimethylthiazol-2-yl)-2,5-diphenyltetrazolium bromide] colorimetric method [21,22].

Cells were harvested during the logarithmic growth phase and seeded in 96-well plates at a density of 1 Å~10^4^ cells/mL, and cultured at 37 °C in a humidified incubator (5% CO_2_) for 24 h, followed by exposure to various concentrations of compounds tested for 48 h. The microtitration colorimetric method of MTT reduction was used in order to identify surviving cells at the end of the treatment period. Subsequently, 20 μL of MTT solution (5 mg/mL) was added to each well and mixed, after which the cells were incubated for an additional 4 h. The solution in each well containing media, unmetabolized MTT and dead cells was removed by suction and 100 μL of DMSO was added. Cell growth inhibition was determined by measuring the absorbance (Abs) at λ = 570 nm using a microplate reader and then calculated. The half maximal inhibitory concentrations (IC_50_) were obtained from liner regression analysis of the concentration-response curves plotted for each tested compound. Stock solutions (5 mg/mL) were prepared by dissolving pure compounds in DMSO and storing them at 4 °C. Serial dilutions with culture media were prepared just prior to the addition to test plates. Doxorubicine was used as the positive control and vehicle (DMSO + Media) as Blank.

#### 3.1.2. Topoisomerase II Decatenation Assay

The kinetoplast-DNA (kDNA) assay was performed according to the protocol of Inspiralis (Norwich, UK). The enzymatic reaction was performed in a total volume of 30 µL containing 200 ng kDNA, test compound (doxorubicine as control or synthesized compounds in DMSO), and 1 unit of topoisomerase II in assay buffer (5 mM Tris.HCl pH 7.5, 12.5 mM NaCl, 1 mM MgCl_2_, 0.5 mM DTT, 10 μg/mL albumin and 1 mM ATP). The mixture was incubated at 37 °C for 30 min and the reaction was terminated by the addition of 5 µL of stop buffer (5% sarkosyl (*n*-lauroylsarcosine, sodium salt), 0.125% bromophenol blue, 50% glycerol). DNA products were analyzed by electrophoresis through 1% agarose gels containing ethidium bromide. kDNA form extremely large networks of high molecular weight that fail to enter an agarose gel. Decatenation of kDNA by topoisomerase II results in minicircular DNAs moving in the gel due to their small size.

### 3.2. Chemistry

#### 3.2.1. General Experimental Procedures

All reactions requiring anhydrous conditions were conducted over flame dried apparatus under an argon atmosphere. THF was freshly distilled from benzophenone-sodium. All reagents and starting materials were purchased from commercial sources and used upon reception. Analytical TLC were carried out on silica gel F254 plates. Visualization was achieved by UV light (254 nm). Flash column chromatography was carried using Sigma-Aldrich Versaflash silica gel (particle size 20–45 μm). Melting points (Mp) were measured on a Büchi B-540 capillary melting point apparatus and are uncorrected. ^1^H and ^13^C NMR spectra were recorded in CDCl3 using a Bruker Avance 300 FT spectrometer at ambient temperature (operating frequencies: ^1^H, 300.13 MHz; ^13^C, 75.47 MHz). The chemical shifts (δ, ppm) for all compounds are listed in parts per million downfield from tetramethylsilane using the NMR solvent as an internal reference. The reference values used for deuterated chloroform (CDCl_3_) were 7.26 and 77.00 ppm for ^1^H and ^13^C NMR spectra, respectively. Multiplicities are given as: s (singlet), brs (broad singlet), d (doublet), t (triplet), dd (doublet of doublets), td (triplet of doublets) or m (multiplet). Low-resolution mass (MS) were recorded on a Shimadzu GCMS-QP 2010 Gas Chromatograph Mass Spectrometer and reported in units of mass to charge (*m/z*). The mode of ionization used was electron-impact (EI, 70 eV) or chemical ionization (CI, methane reagent gas).

#### 3.2.2. Synthesis of Compounds **5**, **6**, **7**, **8**, **9**, **11** and **12**

##### 3,4-Dihydroquinoxalin-2(*1H*)-one (**5**)

To a solution of 1,2-benzenediamine (1 g, 9.26 mmol, 1 eq.) in 20 mL THF under argon atmosphere were added 1.5 mL of ethyl-2-bromoacetate (1.5 mL, 13.51 mmol, 1.46 eq.) and 2 mL of triethylamine (2.0 mL, 14.25 mmol, 1.54 eq.) dropwise. The mixture was heated under reflux for 2 days, before a saturated solution of ammonium chloride was added (30 mL). The crude was extracted with ethyl acetate (3 × 30 mL), the organic phases dried over anhydrous magnesium sulfate, and concentrated *in vacuo*. Purification using (CH/AcOEt) gave the quinoxalinone 5 as a yellowish solid with a 65% yield (0.89 g). Mp 132–134 °C.

^1^H NMR (DMSO, 300 MHz): δ 3.70 (s, 2H, CH_2_), 5.93 (brs, 1H, NH), 6.57 (ddd, 1H, *J* = 7.8, 7.2, 1.5 Hz, H_Ar_), 6.64 (dd, 1H, *J* = 7.8, 1.5 Hz, H_Ar_), 6.71 (dd, 1H, *J* = 5.1, 1.5 Hz, H_Ar_), 6.74 (m, 1H, H_Ar_), 10.22 (brs, 1H, N**H**CO). ^13^C NMR (DMSO, 75 MHz): δ 46.8 (CH_2_), 113.7 (CH_Ar_), 115.4 (CH_Ar_), 118.1 (CH_Ar_), 123.2 (CH_Ar_), 126.6 (C), 135.3 (C), 166.5 (C=O). MS (EI): *m/z* = 119, 100% [M-NHCH_2_], 148, 61% [M].

##### 4-(3-Méthylbut-2-ènoyl)-3,4-dihydroquinoxalin-2(*1H*)-one (**6**)

To a solution of quinoxalinone (**5**) (500 mg, 3.4 mmol, 1 eq.) in 10 mL anhydrous pyridine under argon atmosphere at 0 °C was added 3,3-dimethylacryloyl chloride (0.52 mL, 4.0 mmol, 1.2 eq.) dropwise, and the solution stirred for 2 h. A solution of hydrochloric acid 1N (50 mL) was then added. The product was extracted with ethyl acetate (3 × 30 mL), the combined organic layers dried over anhydrous magnesium sulfate, and concentrated *in vacuo*. Purification by flash chromatography (CH/AcOEt) yielded a beige solid (0.61 g, 78%). Mp 188–190 °C.

^1^H NMR (DMSO, 300 MHz): δ 1.79 (s, 3H, CH_3_), 1.98 (s, 3H, CH_3_), 4.32 (s, 2H, CH_2_), 5.87 (brs, 1H, CHCO), 7.01 (m, 2H, H_Ar_), 7.16 (td, 1H, *J* = 7.8, 1.5 Hz, H_Ar_), 7.26 (m, 1H, H_Ar_), 10.72 (s, 1H, NHCO). ^13^C NMR (DMSO, 75 MHz): δ 20.5 (2 × CH_3_), 26.9 (CH_2_), 116.7 (CH_Ar_), 117.5 (CH_Ar_), 122.5 (C), 124.5 (CH_Ar_), 126.4 (CH_Ar_), 127.1 (C), 131.9 (CH_All_), 151.8 (C), 165.2 (C), 167.8 (C=O). MS (EI): *m/z* = 230, 100,0% [M], 231, 14% [M + 1].

##### 7,7-Diméthyl-6,7-dihydropyrido[1,2,3-*de*]quinoxaline-2,5(*1H,3H*)-dione (**7**)

Under argon atmosphere, quinoxalinone (**6**) (200 mg, 0.9 mmol, 1 eq.) was solubilized in 20 mL of anhydrous DCM. The solution was cooled to 0 °C and aluminum trichloride was added in portions over 1 h (463 mg, 3.5 mmol, 4 eq.). Once the addition finished, the mixture was left to stir for 24 h at room temperature. The crude reaction was then slowly hydrolyzed on ice and extracted with DCM (5 × 30 mL). Organic phases were washed with a saturated solution of sodium bicarbonate and a saturated solution of sodium chloride, dried over anhydrous magnesium sulfate, and concentrated *in vacuo*. The crude was recrystallized in DCM and product (**7**) was obtained as a beige solid with a 83% yield (0.16 g). Mp 211–215 °C.

^1^H NMR (DMSO, 300 MHz): δ 1.22 (s, 6H, 2 × CH_3_), 2.47 (s, 2H, CH_2_), 4.36 (s, 2H, CH_2_), 5.87 (s, 1H, CH_2_), 6.84 (dd, 1H, *J* = 7.2, 2.1 Hz, H_Ar_), 6.99 (m, 2H, H_Ar_), 10.71 (s, 1H, N**H**CO). ^13^C NMR (DMSO, 75 MHz): δ 27.3 (2 × CH_3_), 33.6 (C), 43.9 (CH_2_), 45.1 (CH_2_), 114.6 (CH_Ar_), 118.6 (CH_Ar_), 123.3 (C), 124.4 (CH_Ar_), 127.4 (C), 134.6 (C), 164.6 (C=O), 167.9 (C=O). MS (EI): *m/z* = 215, 100% [M-CH_3_], 230, 96% [M]. C_13_H_14_N_2_O_2_ (230.11): calcd. C 67.81, H 6.13, N 12.17; found C 68.14, H 6.01, N 11.97.

##### 9-Amino-7,7-diméthyl-6,7-dihydropyrido[1,2,3-*de*]quinoxaline-2,5(*1H,3H*)-dione (**8**)

To a solution of compound (**7**) (0.7 g, 3.0 mmol, 1 eq.) in a 20 mL mixture DCM/DMF 1/2 (v/v) at 0 °C was slowly added ultrapure nitric acid 99.99% (0.16 mL, 0.2 mmol, 1.2 eq.). The addition finished, the mixture was left to stir for 24 h at 0 °C. Afterwards, the mixture was added on ice before being extracted with DCM (3 × 20 mL). The combined organic layers were washed with a saturated solution of sodium hydrogen carbonate, dried over anhydrous magnesium sulfate, and concentrated *in vacuo* to obtain the desired nitro compound which is used without purification. The crude was then solubilized in a 10 mL mixture of DMF/DMSO (4/1, v/v) under argon atmosphere, and the solution degassed with argon. Palladium on charcoal was added (0.04 g, 0.2 eq. m/m), and the mixture degassed once more. Hydrogen was then bubbled into the mixture for 18 h and filtered on celite, then concentrated *in vacuo* and lyophilized. After washes with DCM and ethyl acetate, compound (**8**) was isolated with a 29% yield as a beige solid (0.12 g). Mp 257–261 °C.

^1^H NMR (DMSO, 500 MHz): δ 1.16 (s, 6H, 2 × CH_3_), 2.38 (s, 2H, CH_2_), 4.29 (s, 2H, CH_2_), 5.11 (brs, 2H, NH_2_), 6.10 (d, 1H, *J* = 2.0Hz, H_Ar_), 6.24 (d, 1H, *J* = 2.0Hz, H_Ar_), 10.49 (brs, 1H, NH). ^13^C NMR (DMSO, 75 MHz): δ 27.4 (2 × CH_3_), 33.6 (C), 44.1 (CH_2_), 45.5 (CH_2_), 99.6 (CH_Ar_), 104.5 (CH_Ar_), 112.9 (C), 128.1 (C), 135.5 (C), 146.2 (C), 164.9 (C=O), 166.9 (C=O). MS (IE): *m/z* = 245, 100% [M]. C_13_H_15_N_3_O_2_ (245.14): calcd. C 63.66, H 6.16, N 17.13; found C 62.51, H 6.03, N 17.88.

##### 7,7-Dimethyl-1,2,3,5,6,7-hexahydropyrido[1,2,3-*de*]quinoxaline (**9**)

To a solution of compound (**7**) (100 mg, 0.5 mmol, 1 eq.) in 20 mL THF dry under argon atmosphere at 0 °C, was added dropwise a solution of borane/THF complex (2.1 mL, 2.1 mmol, 5 eq.). The addition finished, the solution was then allowed to heat at room temperature for 2 h. The solution was added on ice and extracted with ethyl acetate (3 × 30 mL), washed with a saturated solution of sodium chloride, dried over anhydrous magnesium sulfate, and concentrated *in vacuo*. After purification by chromatography (CH/AcOEt), product (**9**) was obtained as a white oil with a 81% yield (0.07 g).

^1^H NMR (DMSO, 300 MHz): δ 1.18 (s, 6H, 2 × CH_3_), 1.69 (m, 2H, CH_2_), 2.96 (m, 2H, CH_2_), 3.02 (m, 2H, CH_2_), 3.31 (m, 2H, CH_2_), 3.38 (s, 3H, CH_2_), 5.36 (s, 1H, NH), 6.16 (dd, 1H, *J* = 7.2, 1.8 Hz, H_Ar_), 6.38 (m, 2H, H_Ar_). ^13^C NMR (DMSO, 75 MHz): δ 32.0 (C), 32.4 (2 × CH_3_), 37.7 (CH_2_), 40.7 (CH_2_), 46.5 (CH_2_), 48.7 (CH_2_), 110.6 (CH_Ar_), 114.8 (CH_Ar_), 118.1 (CH_Ar_), 130.7 (C), 131.1 (C), 134.8 (C). MS (EI): *m/z* = 202, 100% [M], 147, 47% [M-CHC(CH_3_)_2_]. C_13_H_1__8_N_2_ (202.15): calcd. C 77.18, H 8.97, N 13.85; found C 78.01, H 8.51, N 13.51.

##### 9-(2-Bromophenylamino)-7,7-dimethyl-6,7-dihydropyrido[1,2,3-de]quinoxaline-2,5(*1H*,*3H*)-dione (**11**)

To a solution of 9-amino-quinoxalinone (**8**) (0.15 g, 0.61 mmol, 1 eq.) in 8 mL anhydrous dioxane in a Schlenk flask under argon were added potassium carbonate (1.69 g, 12.24 mmol, 20 eq.), 2-bromoiodobenzene (0.12 mL, 0.92 mmol, 1.5 eq.) and Xant-PHOS (14 mg, 0.02 mmol, 0.04 eq.). The mixture was degassed with argon, and Tris(dibenzilideneacetone)dipalladium (11 mg, 0,01 mmol, 0.02 eq.) was added. The Schlenk flask was sealed and heated to reflux for 18h. The mixture was filtered on celite, added to an ammonium chloride solution (50 mL), and extracted with ethyl acetate (3 × 30 mL). Organic phases were dried over magnesium sulfate, and concentrated *in vacuo*. The desired product was isolated as a beige solid with a 71% yield (0.17 g) after purification by chromatography (CH/AcOEt). Mp 208–210 °C.

^1^H NMR (DMSO, 200 MHz): δ 1.20 (s, 6H, 2 × CH_3_), 2.46 (s, 2H, CH_2_), 4.35 (s, 2H, CH_2_), 6.58 (s, 1H, H_Ar_), 6.75 (s, 1H, H_Ar_), 6.85 (m, 1H, H_Ar_), 7.26 (m, 2H, 2 × H_Ar_), 7.60 (m, 1H, H_Ar_), 10.60 (brs, 1H, NH).^13^C NMR (DMSO, 75 MHz): δ 27.3 (2 × CH_3_), 33.7 (C), 44.0 (CH_2_), 45.2 (CH_2_), 104.0 (CH_Ar_), 109.4 (CH_Ar_), 114.4 (C), 117.0 (CH_Ar_), 119.7 (CH_Ar_), 122.7 (CH_Ar_), 128.0 (C), 128.9 (CH_Ar_), 133.7 (C), 135.6 (C), 140.0 (C), 142.0 (C), 164.7 (C=O), 167.3 (C=O). MS (CI): *m/z* = 401, 100% ([M + 1, ^81^Br], 399, 96% [M + 1, ^79^Br]. C_19_H_1__8_BrN_3_O_2_ (400.28): calcd. C 57.01, H 4.53, N 10.50; found C 56.51, H 4.21, N 10.21.

##### Compound **12**

To a solution of 9-(2-bromophenylamino)-quinoxalinone (**11**) (0.1 g, 0.25 mmol, 1 eq.) in 8 mL DMA in a Schlenk flask under argon were added potassium carbonate (0.10 g, 0.75 mmol, 3 eq.) and palladium (II) acetate (17 mg, 0.07 mmol, 0.3 eq.). The mixture was heated to DMA reflux for 24 h, then filtered on celite, added to a saturated solution of ammonium chloride (25 mL) and extracted with ethylacetate (3 × 15 mL). Organic phases were dried over magnesium sulfate and concentrated *in vacuo*. The final compound (**12)** was isolated as a beige solid with a 40% yield (31 mg) after purification over silica (CH/AcOEt). Mp 308–313 °C

^1^H NMR (DMSO, 200 MHz): δ 1.33 (s, 6H, 2 × CH_3_), 2.55 (s, 2H, CH_2_), 4.52 (s, 2H, CH_2_), 7.15 (m, 2H, 2 × H_Ar_), 7.36 (m, 1H, H_Ar_), 7.47 (m, 1H, H_Ar_), 8.37 (m, 1H, H_Ar_), 10.47 (brs, 1H, NH), 11.33 (s, 1H, CONH). ^13^C NMR (DMSO, 75 MHz): δ 27.5 (2 × CH_3_), 34.1 (C), 44.1 (CH_2_), 45.4 (CH_2_), 101.3 (CH_Ar_), 109.6 (CH_Ar_), 111.0 (CH_Ar_), 117.0 (C), 118.8 (CH_Ar_), 120.8 (C), 121.9 (C), 122.8 (C), 125.5 (CH_Ar_), 134.3 (C), 138.1 (C), 140.2 (C), 165.7 (C=O), 167.8 (C=O). MS (EI): *m/z* = 319, 100% [M], 304, 68% [M-CH_3_]. C_19_H_1__7_N_3_O_2_ (319.13): calcd. C 71.46, H 5.37, N 13.16; found C 70.82, H 5.49, N 13.33.

## 4. Conclusions 

In short, we have developed the synthesis of a new series of marine alkaloids analogues, such as makaluvamine A or neoamphimedine, and have evaluated their potential as antitumor agents. Compounds **9**, **11** and **12** showed activity against different cancer cell lines such as Pc-3 or HCT-116, with no apparent selectivity toward human fibroblast for compounds **11** and **12**. Compound **9** was found to have an interesting profile, having a cytotoxicity of 15, 15, 15 and 10 μM against Caco-2, HCT-116, Pc-3 and NCI cell lines respectively, without toxicity against Human fibroblast. Although, pentacycle **12** was found to have cytotoxic effects on different cancer cell lines (HCT-116, Pc-3 and NCI), it did not express any real selectivity. Nevertheless, its DNA binding capacity showing its potential as a Topoisomerase II inhibitor will allow us to undertake new SAR studies by synthesizing analogues of compound **12** in order to search for more potent activity and better selectivity. 

## References

[1] Newman D.J., Cragg G.M. (2007). Natural products as sources of new drugs over the last 25 years. J. Nat. Prod..

[2] Attmann K.-H., Gertsch J. (2007). Anticancer drugs from nature—Natural products as a unique source of new microtubule-stabilizing agents. Nat. Prod. Rep..

[3] Roberts M., Wink M. (1998). Alkaloïds Biochemistry, Ecology and Medicinal Applications.

[4] Manske R.H.F. (1981). The Alkaloids: Chemistry and Physiology.

[5] Aniszewski T. (2007). Alakaloïds—Secret of Life, Alkaloïd Chemistry, Biological Significance, Applications and Ecological Role.

[6] Meissner C.F.W. (1819). Über Pflanzenalkalien: II. Über ein neues Pflanzenalkali (Alkaloid). J. Chem. Phys..

[7] Deane F.M., O’Sullivan E.C., Maguire A.R., Gilbert J., Sakoff J.A., McCluskey A., McCarthy F.O. (2013). Synthesis and evaluation of novel ellipticines as potential anti-cancer agents. Org. Biomol. Chem..

[8] Ponder J., Yoo B.H., Abraham A.D., Li Q., Ashley A.K., Amerin C.L., Zhou Q., Reid B.G., Reigan P., Hromas R. (2011). Neoamphimedine circumvents metnase-enhanced DNA topoisomerase II alpha activity through ATP-competitive inhibition. Mar. Drugs.

[9] Barrows L.R., Radisky D.C., Copp B.R., Swaffar D.S., Kramer R.A., Waters R.L., Ireland C.M. (1993). Makaluvamines, marine natural products, are active anti-cancer agents and DNA topo II inibitors. Anti-Cancer Drug Des..

[10] Mehbub M.F., Lei J., Franco C., Zhang W. (2014). Marine sponge derived natural products between 2001 and 2010: Trends and opportunities for discovery of bioactives. Mar. Drugs.

[11] Kornprobst J.M. (2005). Substances Naturelles D’origine Marine: Chimiodiversité, Pharmacodiversité, Biotechnologies.

[12] Nag S., Nadkarni D.H., Qin J.-J., Voruganti S., Nguyen T., Xu S., Wang W., Wang H., Velu S.E., Zhang R. (2012). Anticancer activity and molecular mechanisms of action of makaluvamines and analogues. Mol. Cell. Pharmacol..

[13] West L.M., Northcote P.T., Battershill C.N. (2000). Peloruside A: A potent cytotoxic macrolide isolated from the New Zealand marine sponge *Mycale* sp.. J. Org. Chem..

[14] Kokoshka J.M., Capson T.L., Holden J.A., Ireland C.M., Barrows L.R. (1996). Differences in the topoisomerase I cleavage complexes formed by camptothecin and wakayin, a DNA-intercalating marine natural product. Anti-Cancer Drugs.

[15] Lang G., Pinkert A., Blunt J.W., Munro M.H.G. (2005). Discorhabdin W, the first dimeric discorhabdin. J. Nat. Prod..

[16] Schmidt E.W., Harper M.K., Faulkner D.J. (1995). Makaluvamines H–M and Damirone C from the pohnpeian sponge *Zyzzya fuliginosa*. J. Nat. Prod..

[17] Perry N.B., Blunt J.W., Munro M.H.G. (1988). Discorhabdin D, an antitumor alkaloid from the sponges *latrunculia brevis* and *Prianos* sp.. J. Org. Chem..

[18] Guillard J., Bouclé S. (2012). Synthesis of Analogues of Makaluvamine A. Heterocycles.

[19] TenBrink R.E., Im W.B., Sethy V.H., Tang A.H., Carter D.B. (1994). Antagonist, partial agonist, and full agonist imidazo[1,5-*a*]quinoxaline amides and carbamates acting through the GABAA/Benzodiazepine receptor. J. Med. Chem..

[20] Guillard J., Bouclé S. (2011). Synthesis of a New pyrido[3,2-*b*]carbazole as an ellipticine-makaluvamine hybrid. Synthesis.

[21] Yang S.Y., Jia X.Z., Feng L.Y., Li S.Y., An G.S., Ni J.H., Jia H.T. (2009). Inhibition of topoisomerase II by 8-chloro-adenosine triphosphate induces DNA double-stranded breaks in 8-chloro-adenosine-exposed human myelocytic leukemia K562 cells. Biochem. Pharmacol..

[22] Mosmann T.J. (1983). Rapid colorimetric assay for cellular growth and survival: Application to proliferation and cytotoxicity assays. J. Immunol. Methods.

